# Monitoring recombinant protein expression in bacteria by rapid evaporative ionisation mass spectrometry

**DOI:** 10.1002/rcm.8670

**Published:** 2020-02-18

**Authors:** Joscelyn Sarsby, Lynn McLean, Victoria M. Harman, Robert J. Beynon

**Affiliations:** ^1^ Centre for Proteome Research, Institute of Integrative Biology University of Liverpool Liverpool L69 7ZB UK

## Abstract

**Rationale:**

There is increasing interest in methods of direct analysis mass spectrometry that bypass complex sample preparation steps.

**Methods:**

One of the most interesting new ionisation methods is rapid evaporative ionisation mass spectrometry (REIMS) in which samples are vapourised and the combustion products are subsequently ionised and analysed by mass spectrometry (Synapt G2si). The only sample preparation required is the recovery of a cell pellet from a culture that can be analysed immediately.

**Results:**

We demonstrate that REIMS can be used to monitor the expression of heterologous recombinant proteins in *Escherichia coli*. Clear segregation was achievable between bacteria harvesting plasmids that were strongly expressed and other cultures in which the plasmid did not result in the expression of large amounts of recombinant product.

**Conclusions:**

REIMS has considerable potential as a near‐instantaneous monitoring tool for protein production in a biotechnology environment.

## 1 INTRODUCTION

Rapid evaporative ionisation mass spectrometry (REIMS) is an emerging technique for the real‐time analysis of biomolecules.^1^ REIMS is an ionisation technique based on sudden heating of a sample, predominantly using diathermy, generating a plume of aerosol‐containing combustion products. The molecular species in the smoke can be ionised and analysed by mass spectrometry. Most commonly, negative ions derived from fatty acids and phosphoglycerolipids dominate the ensuing mass spectrum.^1^, ^2^ REIMS lends itself extremely well to real‐time surgical analysis owing to the widespread use of electrosurgical instrumentations where an electrical current is applied to the sample resulting in simultaneous cutting and cauterisation. The by‐product is an aerosol and the ions and molecules present in the smoke are indicative of the physiological state and type of tissue. Successful applications include the distinction between healthy and cancerous breast tissue^3^ and malignant gynaecological tissue.^4^ Outside of surgical environments, REIMS has been used for the authentication of food products such as pistachios,^5^ fish,^6^ pork^7^ and beef.^8^

However, the REIMS ionisation process is not limited to tissue samples. REIMS analyses of bacterial and fungal colonies and cultures show promise for the identification of genus and species,^9^, ^10^, ^11^, ^12^, ^13^ correctly identifying up to 28 clinically relevant bacterial and fungal species with an accuracy of up to 99% when predicting the gram stain result and at 88% accuracy when identifying specific species. Recent developments include robotic colony identification and sampling to enable automated high‐throughput methods for bacterial analysis.^10^, ^14^

REIMS generates complex spectra that are not readily deconstructed into the identities of the constituent molecules. Thus, a REIMS data file is analysed as a ‘binned’ mass spectrum over a broad mass range that is treated as multivariate data. To gain biological meaning from REIMS data, statistical methods such as principal component analysis (PCA), linear discriminant analysis (LDA) and random forests are regularly employed.^15^ Each of these approaches assesses the entire binned data file data to discover patterns and similarities among common samples and differences between unrelated samples. Accuracy and precision are determined via test sets and leave‐one‐out cross‐validation.

To date, all REIMS applications have emphasised the identification of a sample, based on prior learning of known samples in a teaching set. In this study, we assessed the potential of REIMS analysis to track the expression and production of recombinant proteins in *Escherichia coli*. Mass spectrometry has been used extensively to monitor the recombinant protein expression but these approaches have all been based on analysis of the protein itself, whether as an intact protein^16^ or after proteolytic fragmentation.^17^ We have used REIMS to monitor the expression of a series of QconCATs, artificial ‘designer’ proteins that are concatenations of tryptic peptides used in the absolute quantification of proteins. Typically, QconCATs, coded within an expression plasmid, such as pET21a, are produced as 70–90 kDa proteins and are assemblies of tryptic peptides from around 25 target proteins. Because each peptide is released in stoichiometrically identical amounts after tryptic digestion, QconCATs are highly efficient ways to create a pool of approximately 50 peptides for quantification of, typically, 25 proteins.^18^, ^19^, ^20^, ^21^, ^22^ We have observed that QconCAT expression is variable, such that some are expressed at extremely high levels but others are not generated in detectable or useable amounts – a consequence of the design and expression of these highly atypical artificial proteins. This variable degree of expression, and the fact that the QconCATs have no intrinsic biological activity that could directly impact the host cell metabolism, means that they are ideal candidates to explore the use of REIMS to monitor recombinant protein expression.

## 2 EXPERIMENTAL

### 2.1 Bacterial cultures

A total of 22 glycerol stocks of *Escherichia coli* (BL21 *λDE3*) harbouring pET21a plasmids containing different QconCAT genes were used for this study. Details of each QconCAT have been previously reported. Of the QconCATs used here, eight were strong expressors (CC001, CC002, CC015, CC039, CC041, CC091, CC104, CC105), 11 were non‐expressors (CC016, CC024, CC046, CC057, CC062, CC064, CC068, CC069, CC093, CC103) and three were considered to express QconCATs in moderate amounts (CC003, CC092, CC096).^18^ Colonies were grown on LB agar with 50 μg/mL ampicillin at 37°C overnight. Single colonies were used to inoculate 10 mL of LB medium with 50 μg/mL ampicillin, grown overnight at 37°C 140 rpm; this starter culture was subsequently used to inoculate 200 mL of LB with 50 μg/mL ampicillin, again incubated at 37°C 140 rpm. The turbidity of the medium was monitored at 600 nm and at an optical density (OD_600_) of 0.6, and expression of the QconCAT was induced by addition of isopropyl thiogalactoside (IPTG) to a final concentration of 1 mM. Before and after induction, samples of the culture (10 mL) were removed for analysis.

### 2.2 Cell processing

Cells were centrifuged at 13,500 *g* at 4°C, for 15 min and washed with 10 mL of 0.15 M NaCl. Portions of cell pellets, each containing 2.5 × 10^10^ cells, were stored at −20°C until analysis. Expression of recombinant proteins was assessed by sodium dodecyl sulphate polyacrylamide gel electrophoresis (SDS‐PAGE).

### 2.3 REIMS analysis

**FIGURE 1:**
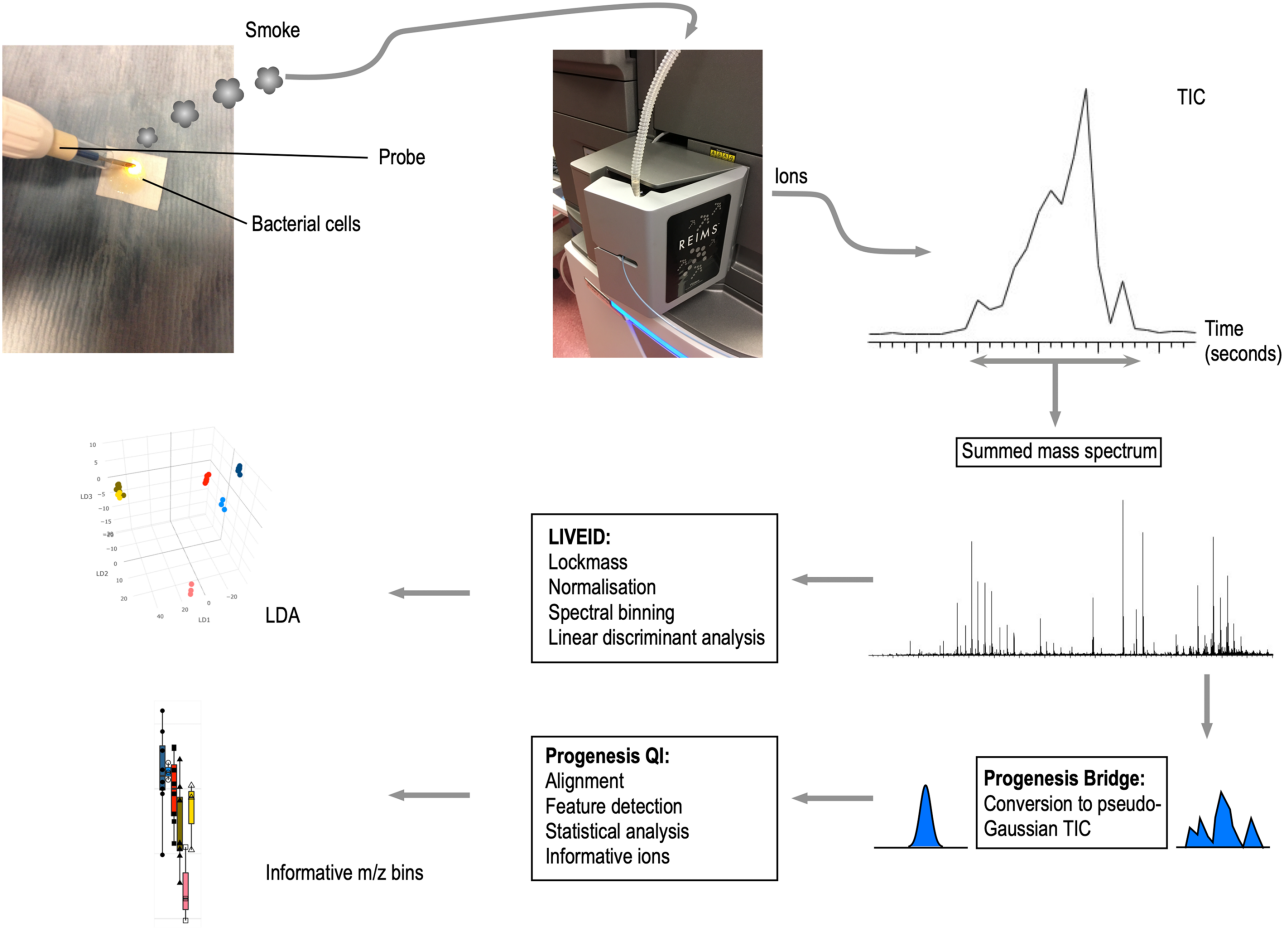
Overview of the workflow for the application of REIMS to monitor heterologous bacterial protein expression [Color figure can be viewed at wileyonlinelibrary.com]

An overview of the REIMS workflow is provided in Figure 1. Cell pellets were thawed and re‐suspended in 100 μL of HPLC‐grade water and pipetted onto glass *microfiber* paper (GFP, GE Healthcare Whatman, Little Chalfont, UK). Sampling used a monopolar diathermy electrosurgical pencil (Erby Medical UK Ltd, Leeds, UK). A power of 10 W was applied across the cell pellet, and the smoke was aspirated and directed into the REIMS source (Waters, Wilmslow, UK). At the same time, a lockmass solution of 5 pmol/μL leu‐enkephalin in propan‐2‐ol at a flow rate of 50 μL/min was delivered to the REIMS source attached to a Synapt G2‐Si mass spectrometer (Waters) in resolution mode. Spectra were recorded in negative ion mode from *m/z* 50 to 1200 at a scan rate of 1 Hz. Burn events lasted from 10 to 30 sec. Spectra were averaged over the entire burn event.

### 2.4 Data processing

RAW data was pre‐processed using Progenesis Bridge, a feature within the Waters MassLynx Software, with a threshold of 0, to normalise the TIC trace and combine the burn events enabling the comparison of a single spectrum from each pellet. These data were processed in LiveID (Waters) for LDA analysis, using lockmass correction and background subtraction. Data were collected from *m/z* 50 to 1200 and subsequently binned with an *m/z* window of 0.01 units. Linear discriminant analysis (LDA) was performed within LiveID. LDA components were calculated using 20 principal components for the data set shown in Figure 4, or 25 principal components for all other data sets. In all cases, the number of LD components was set to one fewer than the number of groups. The pre‐processed data were also imported into Progenesis QI where the raw data were aligned, normalised and peak picked. For peak picking a maximum charge state was set at 1. Statistical analysis was performed to extract the most influential features within the data set that contributed to the separation. This information was combined with the processed data from LiveID, exported as a matrix of sample vs *m/z* bin, and populated with normalised ion intensities. Ion specific data were visualised in R, using RStudio.^23^ Datafiles are available locally at https://www.liverpool.ac.uk/pfg/Research/datafiles/Datafiles.html and at the Metabolights [https://www.ebi.ac.uk/metabolights/studies] data site with accession number MTBLS957.

## 3 RESULTS AND DISCUSSION

Liquid bacterial cultures are not intrinsically compatible with REIMS ionisation whereby a diathermy electrode has to be applied to a hydrated solid or semi‐solid sample. To avoid inclusion of signal from the medium, and to ensure a concentrated burn, bacterial suspensions were first washed with saline, collected by centrifugation and the bacterial pellet was placed onto the centre of a glass microfibre disc (Figure 1). This gave a discrete aggregate of cells, approximately 5 mm across, that could be readily burned by the REIMS probe. Typically, the cell pellet was consumed in a single burn event. Negative ions generated by the burn event were acquired from *m/z* 50 to 1200. In practice, uniform burn events were difficult to obtain as the sample adhered to the electrode, resulting in a segmented total ion current (TIC) trace. To avoid pseudoreplication caused by these discrete segments being considered as separate analyses, the raw data were first processed using Progenesis Bridge to convert the uneven burn events into a single pseudo‐Gaussian (time, TIC) curve. The processed TIC trace was always 35 scans across, with the burn event covering 12 scans reaching a maximum at scan 18. This processed TIC trace was then imported into LiveID ensuring that there was only one datum per burn event in all subsequent analysis.

Having established that this processing workflow was suitable to generate a detailed mass spectrum, we explored the potential of REIMS to monitor the changes attendant upon recombinant protein expression, specifically the expression of QconCATs. QconCATs are artificial proteins, built as assemblies of tryptic peptides from multiple target proteins for selected reaction monitoring (SRM) quantification. Although they have some unique properties,^24^ being more homogeneous in amino acid composition and length than typical *E. coli* proteins, their lack of intrinsic biological activity was an advantage here, as they could not have individual pleiotropic effects. QconCATs do not have higher order structure, and typically aggregate in inclusion bodies, a common observation in heterologous protein expression.

**FIGURE 2:**
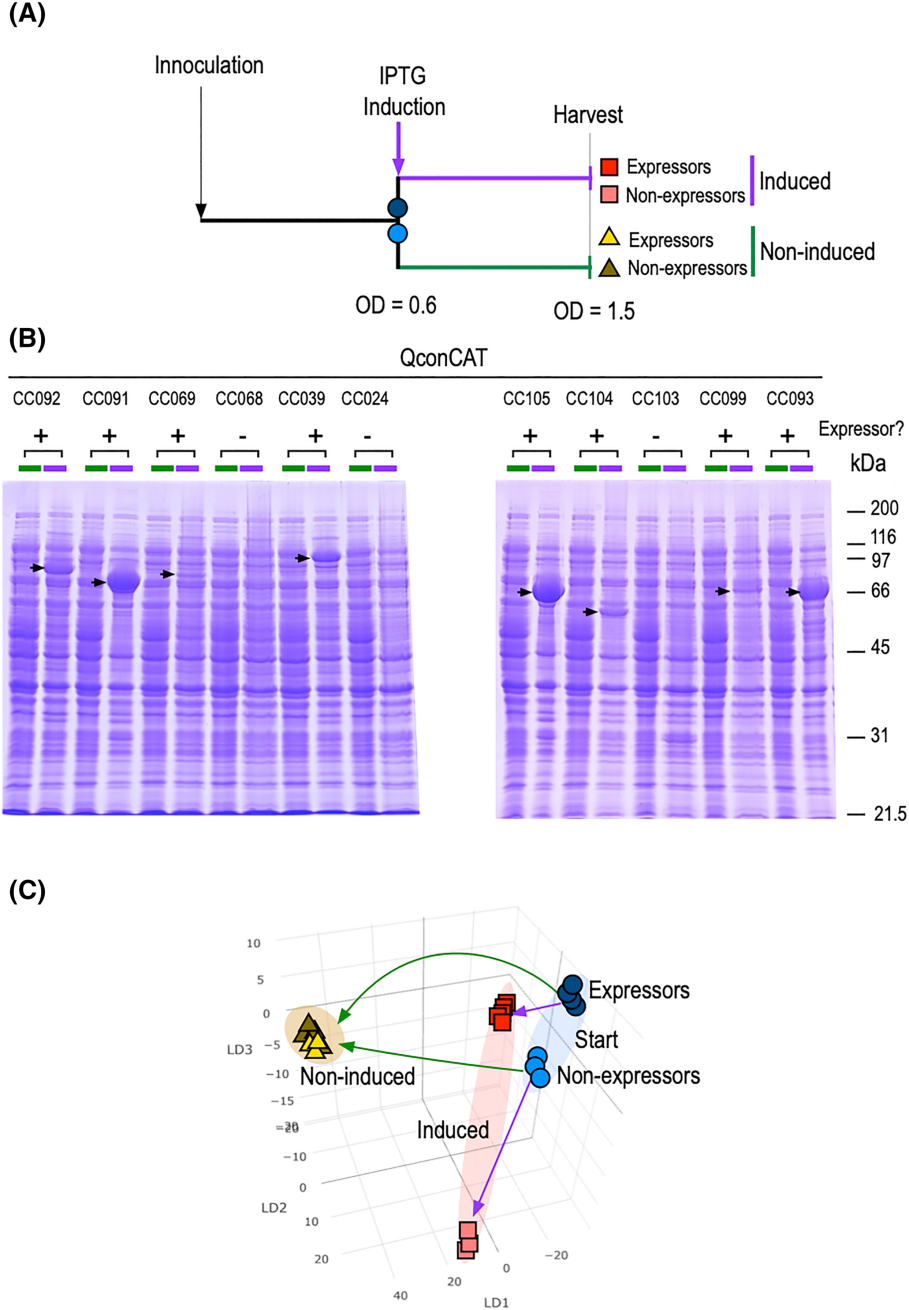
Analysis of recombinant protein expression by REIMS of total cell pellets. Eleven different bacterial strains, each harbouring a plasmid encoding a different QconCAT, were grown in liquid culture. At a cellular density equivalent to an OD_600_ of 0.6, the culture was split into two; in one half expression was induced by addition of IPTG, in the other half growth continued in the uninduced condition to an OD_600_ of 1.5 (A). Expression of QconCAT was assessed by SDS‐PAGE before and after induction (B). Cell pellets were recovered and analysed by REIMS. The aligned, normalised and binned data were then used to drive LDA, and the different conditions are mapped to the first two LDA components (C) [Color figure can be viewed at wileyonlinelibrary.com]

Initial studies monitored the expression of 11 QconCATs, with varying degrees of expression (Figure 2). In some (CC091, CC105 and CC093) expression was high, whereas in others expression was modest (CC092, CC069, CC104, CC099) or not detectable (CC068, CC103). We collected the same number of bacterial cells immediately before induction and after induction of expression. Control incubations (lacking addition of the inducer) were grown to the same OD_600_ as the induced samples, to allow for culture ageing and nutrient depletion. The growth curves (not shown) indicated that the growth rates of all cultures, whether or not induced, were very similar, obviating QconCAT induced toxicity.

From the LDA, several observations emerge. First, based on the classification of expression from SDS‐PAGE (Figure 2B), there is a clear segregation between cells that express proteins and those that do not. However, it was also possible to discriminate the cultures prior to induction. This is curious, as both contain the ampicillin resistance gene and, in the absence of induction, should impose similar metabolic demands on the cells. This warrants further investigation. After induction (Figure 2C) the expressors and non‐expressors deviated very much more in the LDA plot. Finally, in the absence of induction, all cultures moved to a similar position in the LDA space, presumably reflecting the metabolic shift commensurate on continued growth and metabolism. However, the marked segregation of expressors and non‐expressors after induction is clear evidence that the formation of heterologous proteins leads to altered metabolic states that can be resolved by REIMS.

To establish the validity of the data‐processing approach, we repeated the analyses after randomising the sample assignment and re‐running the processing methods. After randomisation, the different cultures gave little evidence of segregation (supporting information). The inability of LDA to create clusters when the input data had been randomised lends confidence to the clustering of the correctly allocated data. It is therefore likely that REIMS can discriminate stage of growth, induction and expression. Although the expressed proteins were non‐physiological and distinct for each culture, the co‐clustering of cultures with similar expression levels was also encouraging.

**FIGURE 3:**
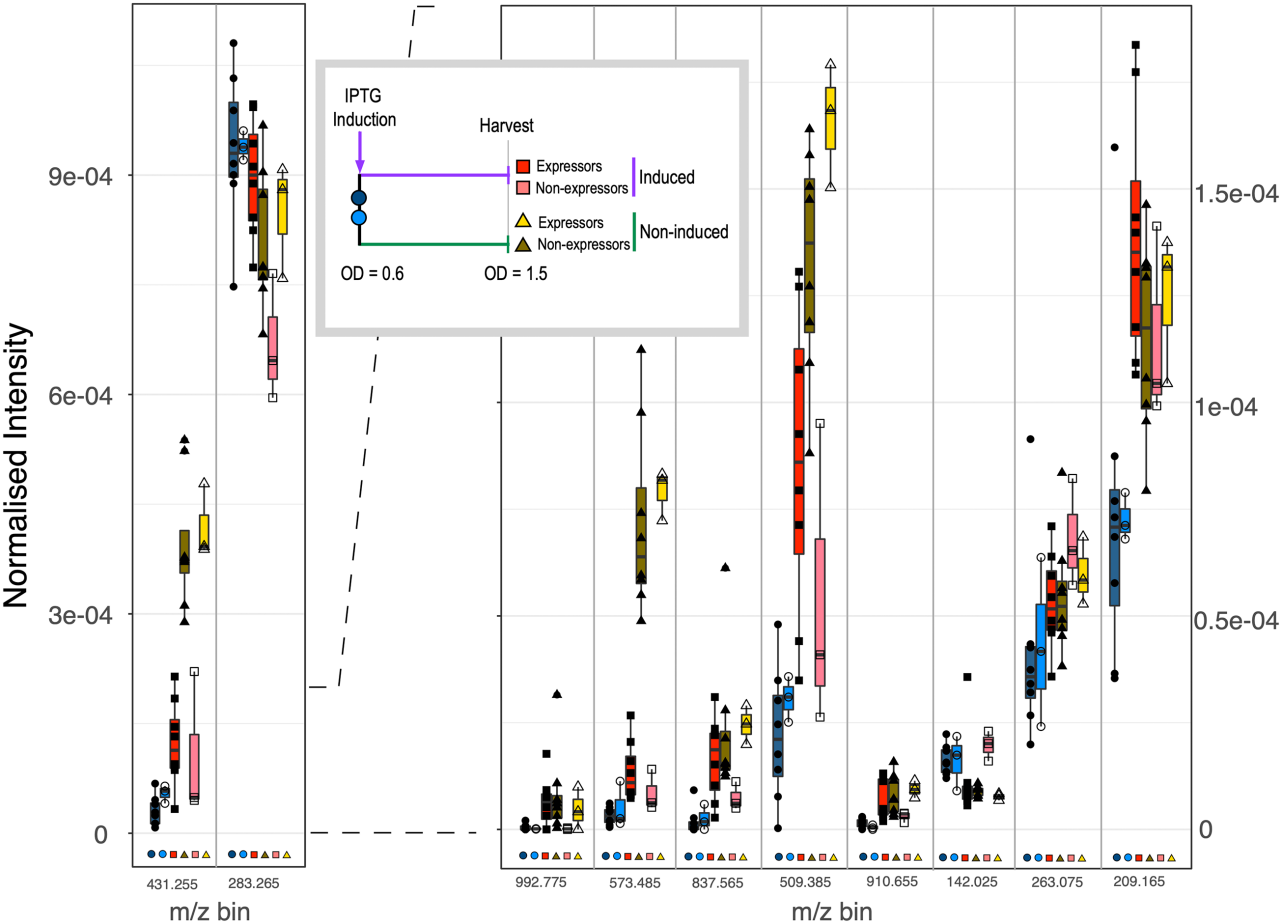
Comparative intensities of informative ions. For the bacterial cultures analysed in Figure 2, the top 10 most informative ions/bins were assessed and displayed as box plots (median, 25^th^ and 75^th^ percentiles, maximum and mininmum, with outliers). Symbols and samples are the same as those in Figure 2

Importing the data into Progenesis QI through Progenesis Bridge allowed us to interrogate the data at a feature level (Figure 3) and focus on specific informative ions/mass bins. Discriminatory spectral features covered the full *m/z* range, encompassing a broad range of intensities. Although the molecular identity of the individual ions is not known, it is clear from this analysis that the two start times (expressors and non‐expressors) are very similar, with significant overlap of intensity values for all ions presented. After induction, there were marked differences in ion intensities, for example, at *m/z* bin 573.485, where there was no overlap between the induced and non‐induced samples, but little or no discrimination between expressors and non‐expressors. Other features, such as at bin *m/z* 283.265 (putatively stearic acid), show a more subtle change, increasing in intensity as expressors are induced, but decreasing in intensity as non‐expressors are induced. Certainly, it is not possible to focus on a few ions that are strongly diagnostic of heterologous protein expression, and discrimination is based on comparative analysis of the complex patterns of ions across the entire range of different *m/z* bins.

**FIGURE 4:**
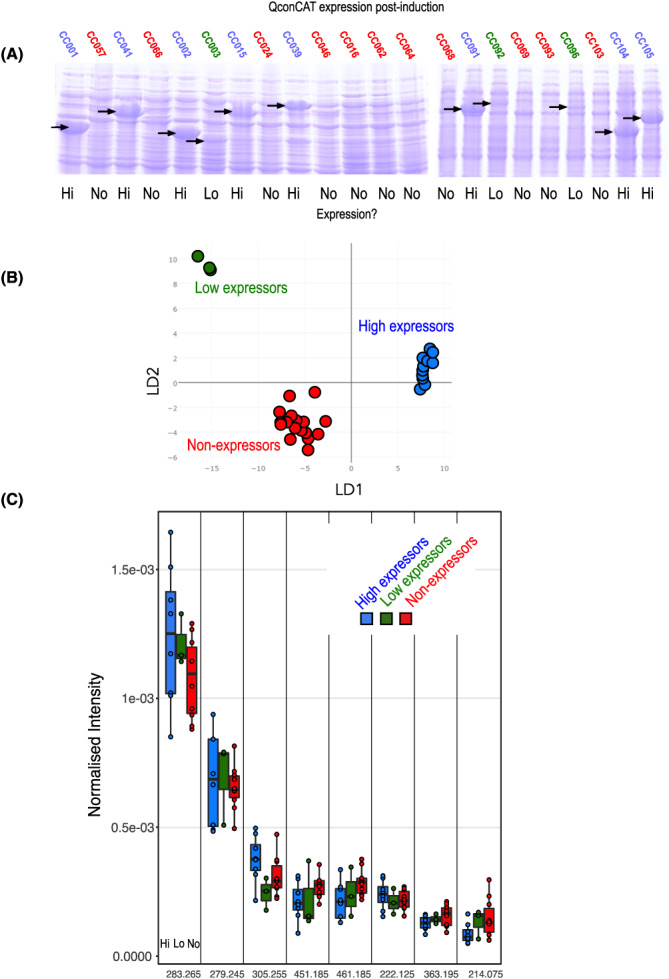
Analysis of degree of heterologous protein expression in *E. coli* by REIMS. A total of 22 different bacterial strains harbouring plasmids encoding different QconCATs were grown to a turbidity corresponding to an OD_600nm_ of 0.6, at which point the inducer was added. After 3 h for continued growth, cells were harvested and analysed for protein expression by SDS‐PAGE (A) or by REIMS. LDA revealed clear separation of the three classes (B), although the changes in the intensities of specific *m/* *z* bins were subtle (C) [Color figure can be viewed at wileyonlinelibrary.com]

As a further test of the feasibility of using REIMS to assess heterologous protein production, another experiment was completed to assess 22 samples, 11 expressors and 11 non‐expressors, analysed post‐induction (Figure 4). The 22 strains were classified (Figure 4A) as being High (‘Hi’, 8), Low (‘Lo’, 3) or non‐expressors (‘No’, 11), based on the appearance of the QconCAT protein band on SDS‐PAGE analysis. REIMS analysis of cell pellets from all 22 strains revealed, through LDA, a clear separation among the three classes (Figure 4B). The resolution of high and low expressors implies that REIMS can not only resolve expressors and non‐expressors, but also assess the extent of protein expression, reflecting perhaps the intensity of the metabolic demand that is created post‐induction. Again, this is reflected in subtle patterns of expression change for different *m/z* bins (Figure 4C). From these data, it seems highly likely that REIMS could provide a rapid assessment of the strength of expression of heterologous proteins.

**FIGURE 5:**
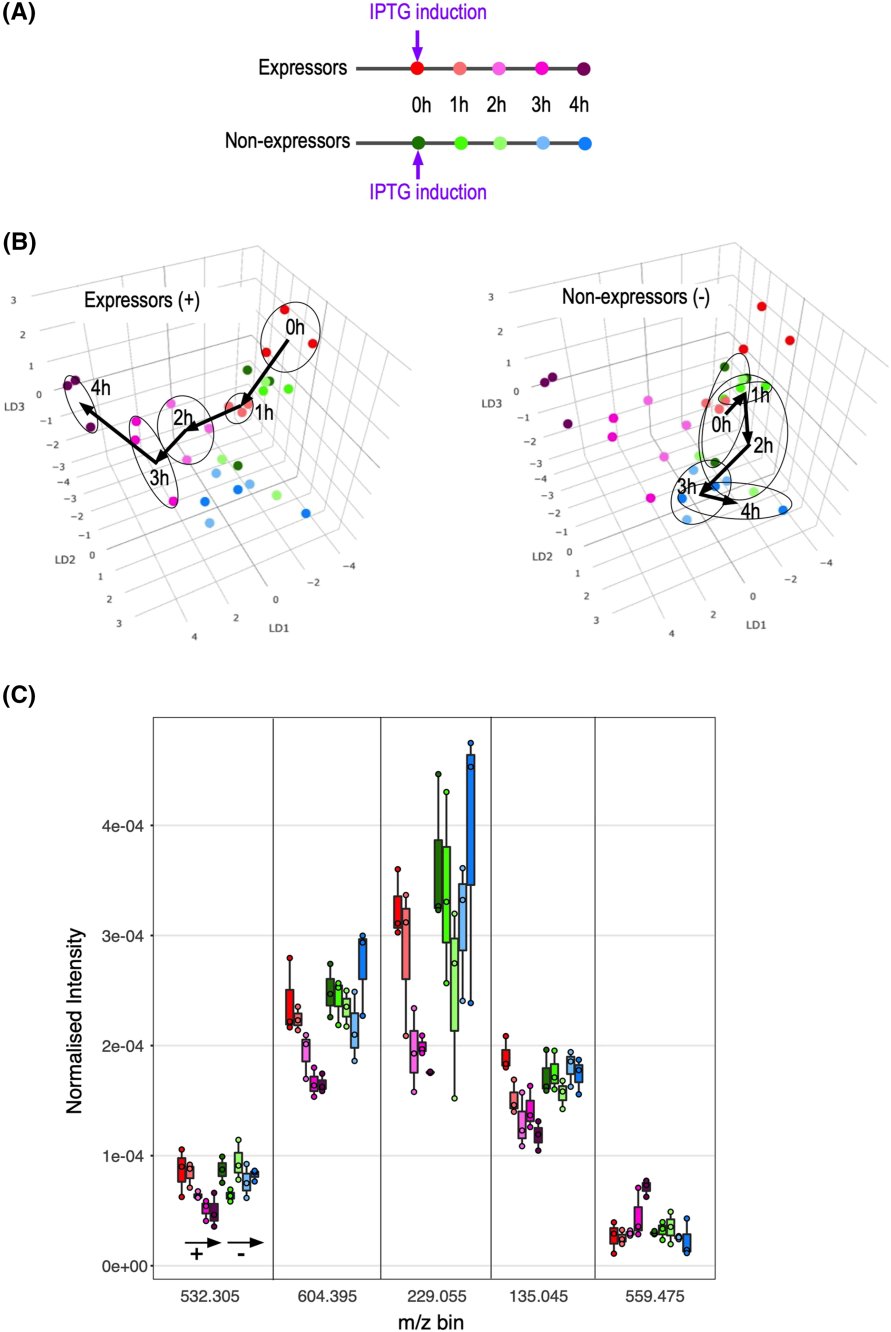
Use of REIMS to track the time course of recombinant protein expression. Six bacterial cultures, three of which contained strongly expressing QconCAT encoding plasmids and three of which contained QconCAT plasmids that failed to express were grown to a turbidity of OD_600_ of 0.6, at which point induction was initiated (A). Samples were removed from each culture at hourly intervals for analysis by REIMS. The paths traced in the three‐component LDA plots differed for expressors (B, left, red to purple) and non‐expressors (B, right, green to blue); note that all data are present in each plot, but that high and low expressors are differentially highlighted in each sub‐panel. The intensities of five informative *m/z* bins that change during induction are plotted in C [Color figure can be viewed at wileyonlinelibrary.com]

In a biotechnology context, it would be optimal to be able to monitor the expression of recombinant proteins over time. REIMS was therefore used to monitor the time‐dependent changes in signals for both induced expressors and non‐expressors (Figure 5). Accordingly, three strong expressors (CC091, CC093, CC105) and three non‐expressors (CC024, CC099, CC103) were established in liquid culture and, after induction, samples were removed at hourly intervals (Figure 5A). It was evident that the shift in the LDA coordinates was progressive, and that the coordinates tracked different paths through LDA space for expressors and non‐expressors (Figure 5B). Analysis of the dominant informative *m/z* bins once again revealed complex patterns (Figure 5C). For example, the ion current centred at *m/z* 229.055 exhibited a progressive decline in intensity in expressors, but a U‐shaped progression for non‐expressors. By contrast, the signal centred at *m/z* 559.475 increased slightly during the induction phase for expressors, and decreased slightly for the non‐expressors. This reiterates that specific ions are unlikely to be uniquely informative, and that the value of REIMS lies in the generation of patterns of ions that can be learned and compared.

These studies provide proof of principle that REIMS analysis, with our current protocol, is capable of detecting subtle changes within a bacterial strain during growth, and that this method of analysis is sensitive to, and can inform on, the diversion of metabolites into the production of recombinant proteins. Furthermore, REIMS may be able to track the extent of expression, and the time‐dependent trajectory of protein production. Further validation of the approach should expand the number of test systems, and include a range of recombinant proteins, bacterial species and the evolution of the REIMS signature over the time course of expression. For example, large‐scale protein production facilities would generate hundreds of samples that would allow testing of the initial data presented here In turn, this would allow exploration of robust analytical approaches, using separate training and validation sets. REIMS analysis would be of value in, for example, large‐scale screening of expression trials. The slowest step in the processing of samples lies in the generation of a REIMS‐compatible bacterial cell pellet that can be analysed. Direct analysis of the culture medium would undoubtedly accelerate the analysis even more. A key feature of REIMS analysis is the generation of patterns of ions that are not interpreted to create molecular identities for specific ions. Undoubtedly, knowledge of the metabolites that are changing during induction of expression could provide new insights into the molecular responses to the added demand of over‐expression of a single protein, but this was beyond the scope of the present study, and would be best explored in a formal metabolomics context.

## 4 CONCLUSIONS

REIMS analysis of cells recovered from liquid bacterial cultures generates informative negative ion mass spectra. The spectra can be used to monitor bacterial growth but, more specifically, can track and monitor the induced expression of recombinant, heterologously expressed proteins. The speed of the analysis suggests that REIMS has value for routine monitoring in biotechnological applications.
